# A novel integrated dressing to secure peripheral intravenous catheters in an adult acute hospital: a pilot randomised controlled trial

**DOI:** 10.1186/s13063-018-2985-9

**Published:** 2018-10-30

**Authors:** Nicole Marsh, Emily Larsen, Jodie Genzel, Gabor Mihala, Amanda J. Ullman, Tricia Kleidon, Sue Cadigan, Claire M. Rickard

**Affiliations:** 1 Royal Brisbane and Women’s Hospital, Herston, QLD Australia; 2 School of Nursing and Midwifery, Griffith University, Nathan, QLD Australia; 3 Alliance for Vascular Access Teaching and Research Group, Menzies Health Institute Queensland, Brisbane, QLD Australia; 4 Lady Cilento Children’s Hospital, Brisbane, QLD Australia; 5 School of Medicine, Griffith University, Brisbane, QLD Australia; 6 Centre for Applied Health Economics, Menzies Health Institute Queensland, Brisbane, QLD Australia

**Keywords:** Randomised Controlled Trial, Occlusive Dressings, Catheterisation, Peripheral, Catheter-Related Infections

## Abstract

**Background:**

The reported incidence of peripheral intravenous catheter (PIV) failure has been as high as 69%. This is in part due to inadequate stabilisation or securement to the skin, which allows micro-motion of the catheter within the vein.

**Methods:**

A pilot open randomised controlled trial of 300 patients was conducted in the medical and surgical wards of a large tertiary hospital. A superiority parallel pragmatic design was used. Eligible patients over the age of 16 years were randomised using a centralised service (randomly varied block sizes and 1:1 ratio) to have PIV dressings of either (i) a bordered polyurethane dressing (BPU, standard care) or (ii) the integrated securement device (ISD). Allocation was concealed until entry. The primary outcome of feasibility addressed eligibility, consent, protocol adherence and retention rates. All-cause PIV failure was an additional primary outcome. This was a composite of infection (laboratory-confirmed local or bloodstream infection), occlusion or infiltration, dislodgement, phlebitis and thrombosis. Group comparisons were by proportions, incidence rates per 1000 PIV days and hazard ratios. Secondary outcomes were local or bloodstream infection, occlusion or infiltration, dislodgement, phlebitis, thrombosis, PIV dwell time, safety and adverse events and patient satisfaction with study products. Analysis was by intention to treat and the patient was the unit of measurement. Multivariable modelling was undertaken.

**Results:**

Feasibility outcomes were 91% of screened patients were eligible, 98% of invited patients consented, 100% of randomised participants received the allocated intervention on insertion and 1/300 (< 1%) were lost to follow-up. In total, 792 PIV days were studied. PIV failure occurred in 43/150 BPU patients (29%) and 40/150 ISD patients (27%) (119 vs 93 per 1000 PIV days; incidence rate ratio 0.78, 95% confidence interval, CI 0.50–1.23). In the multivariate model, ISD (hazard ratio 0.51, 95% CI 0.29–0.89) and admission for a surgical emergency were significantly associated with decreased failure, while female gender, wound, hand insertion and more frequent PIV use were significantly associated with increased PIV failure.

**Conclusion:**

ISDs were significantly associated with decreased failure in the multivariable modelling. Feasibility outcomes were supportive of the need to undertake a larger trial to confirm these results.

**Trial Registration:**

Australian New Zealand Clinical Trials Registry, ACTRN12616000984493. Registered 27 July 2016.

## Background

Peripheral intravenous catheters (PIVs) are the most commonly used vascular access device with approximately 330 million sold each year in the USA alone [1]. Licensed for 29 days use, they are ideally suited for short-term delivery of intravenous fluids or medication [2]. However, it is well documented that PIVs often fail before the completion of intravenous treatment [3–5], with incidence as high as 69% [4, 6–8]. This failure is, in part, a result of inadequate stabilisation or securement of the catheter to the skin [9, 10]. A poorly secured PIV results in micro-motion of the catheter within, or in and out of, the vein [5], leading to partial or complete dislodgement of the catheter; phlebitis or irritation to the vessel wall; infiltration of fluids into the surrounding tissue; occlusion or blockage of the catheter; and in extreme cases, local or systemic infection as skin bacteria are pushed into the wound [6].

The ideal PIV dressing should cover the insertion site, keeping it dry and clean, be comfortable for the patient, as well as offer protection from external contamination and trauma [11]. It should secure the catheter to the skin and stabilise the PIV hub to minimise catheter movement. PIV dressings should be cost-effective, easy to remove and comfortable for the patient [12, 13]. Traditionally, PIV dressings alone were considered to provide adequate catheter securement and did not consider the necessity of *dressing* and *securement* as two separate, but related, needs. In more recent times, international guidelines recommend both the covering of PIVs with a polyurethane dressing to enable clear visualisation of the insertion site [14], as well as the use of an additional securement device to prevent PIV micro-motion and device failure [15]. Engineered stabilisation devices have been designed to offer additional anchor points using a strong adhesive base pad to hold the PIV in place [15, 16]. These devices are used in conjunction with a simple or bordered transparent dressing. However, there is concern that they may have a higher height profile under the dressings, resulting in a tenting effect with possible resultant contamination of the insertion site or increased likelihood of catching on bedding or clothing and dislodging [17].

In addition to stand-alone engineered stabilisation devices, several new dressing and securement options are now available. However, there has been a lack of formal evaluation of these products to assess their efficacy to prevent failure. One such product combines dressing and securement device functions into an integrated securement device (ISD). By combining these functions, a low height profile is possible, reportedly overcome the tenting effect. ISDs are yet to be formally evaluated in a randomised controlled trial (RCT).

### The study

**Fig. 1:**
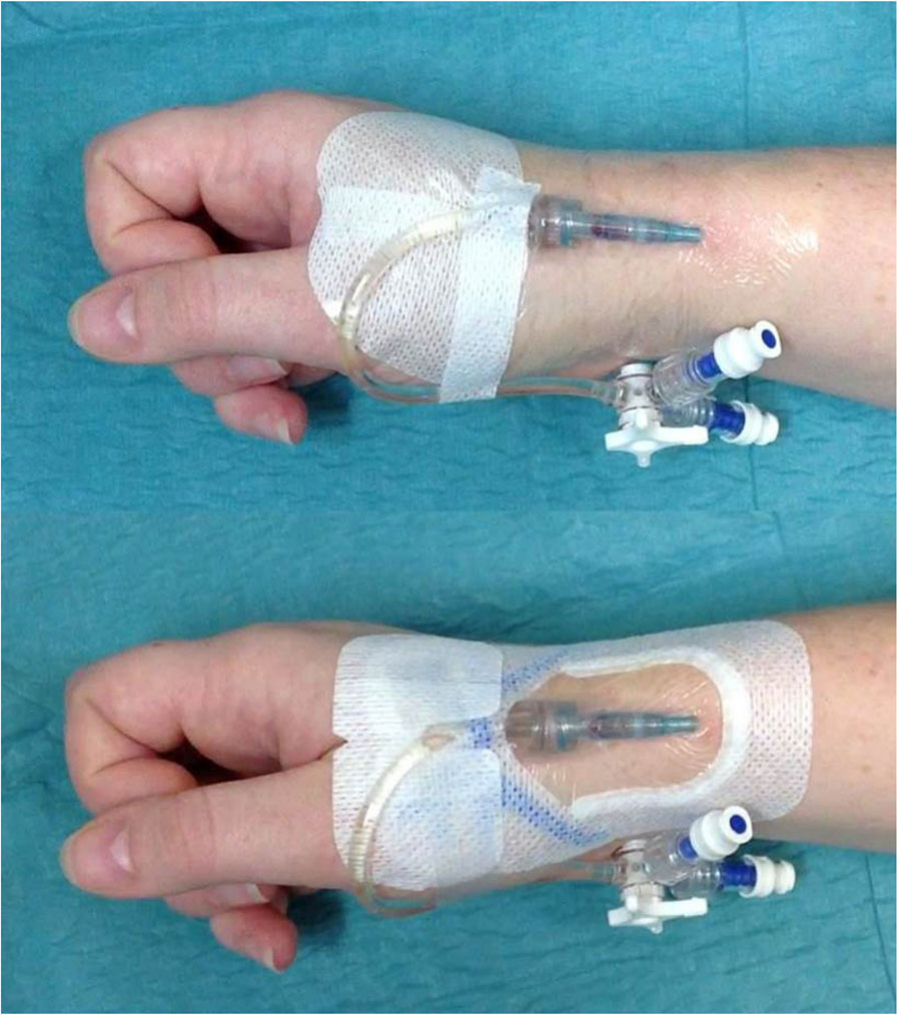
Bordered transparent dressing (top), integrated securement device (bottom)

We compared standard care (bordered polyurethane dressing or BPU, 3M Tegaderm™ 1635) with an ISD (Sorbaview SHIELDTM -SV233, Centurion Medical Products, Williamston) (Fig. 1). This study had two aims:

1. To compare usual care with a novel method of securement
2. To establish the feasibility of the products, protocol and processes to inform the development of a larger definitive RCT.

## Methods

### Study design and participants

We conducted this pilot RCT in a large tertiary hospital in Queensland, Australia. Ethical approval was obtained from Queensland Health (HREC/16/QRCH/75) and Griffith University (2016/487). The trial was registered with the Australian New Zealand Clinical Trials Registry (ACTRN12616000984493). Patients in the medical and surgical wards were screened by a research nurse (ReN) at least every second day between October 2016 and July 2017. Patients were eligible to participate in the trial:

- if they were over the age of 16
- if they were having a PIV inserted with an expected use of greater than 24 h
- if they provided written and informed consent.

Patients were excluded from this study:

- if they had a current bloodstream infection (within 48 h)
- if they had previously participated in the trial
- if they did not speak English and required an interpreter
- if they had other types of vascular access devices
- if their skin was burned or scarred at the insertion site
- if their skin was papery or at risk of a skin tear
- if they had a known allergy to the study products.

### Sample size calculation

For our pilot RCT, the recruitment target was 150 participants per group. This sample size was not chosen to achieve a level of statistical power, but rather to establish the availability of study participants, protocol adherence and acceptability of dressings for a larger adequately powered RCT [18].

### Randomisation and masking

Once a patient had consented to participate in the study, an ReN accessed a web-based centralised randomisation service provided by Griffith University to obtain the group allocation. Patients were randomly assigned to a 1:1 ratio with computer-generated and randomly varied block sizes of two and four to prevent allocation prediction. Due to the nature of the trial, it was not possible to mask clinical staff or the ReN to the dressing allocation. However, an infectious diseases physician was blinded to the group allocation when assessing the secondary outcome of laboratory-confirmed bloodstream infection.

### PIV care and maintenance

PIVs were inserted by experienced vascular access nurses guided by the local hospital policy. The skin was prepared with SoluPrep™ Antiseptic Swab (2% chlorhexidine in 70% isopropyl alcohol; 3M, St Paul). All PIVs were Insyte™ Autoguard™ blood control (non-winged) catheters (Becton Dickinson, Utah). A Smart-Site™ needle-free valve (Becton Dickinson) was added directly to 10 cm of extension tubing, which included a bonded three-way connector (Connecta™, Becton Dickinson). The allocated product was applied to the PIV at insertion. Dressing products were left at the patient’s bedside so that bedside nurses could replace dressings that were loose or soiled. The PIVs were cared for as per usual practice by bedside clinical staff. Any decision to remove a PIV and collect skin swabs, catheter tips or blood cultures was that of the treating clinician, not the investigators.

### Outcome measures

The first primary outcome for this study was feasibility measured as patient eligibility, recruitment (consent), protocol adherence and retention. The second primary outcome was all-cause PIV failure, which was a composite of infection (laboratory-confirmed local or bloodstream infection), occlusion or infiltration (includes leaking), dislodgement, phlebitis and thrombosis (suspected or confirmed).

### Secondary measures

The secondary outcomes were PIV failure due to:

- infection (laboratory-confirmed local venous or primary bloodstream infection) [19]
- occlusion (when the PIV will not infuse) or infiltration (movement of fluids into the surrounding tissues) [4, 20]
- dislodgement (complete or partial dislodgement of catheter) [11]
- phlebitis (as per clinician decision or the presence of two or more of pain, redness, swelling or a palpable cord) [3]
- thrombosis (suspected as assessed by treating clinician or confirmed by medical imaging)
- PIV dwell time (from insertion until removal)
- safety, measured as serious adverse events and adverse skin events potentially related to dressing and securement (e.g. itching, rash, blister, skin tear, bruising and pressure areas)
- patient acceptability of the study products (11-point scale, from 0 = dissatisfied to 10 = satisfied).

We initially also planned to collect staff satisfaction but did not proceed with this since products rarely needed replacement and not enough nurses applied the ISD product to provide meaningful data.

### Data collection

The data for this study were collected by a ReN and entered into an electronic data platform supported by REDCap™ (Research Electronic Data Capture 6.10.6 © 2016 Vanderbilt University). At recruitment, demographic information (e.g. admitting diagnosis, age, sex, co-morbidities and skin integrity) and PIV data (e.g. catheter gauge, site of insertion and number of insertion attempts) were collected. Following PIV insertion, information about the dressing was collected (whether the patient received the correct dressing and whether additional dressing products were applied). The PIV site was inspected daily by the ReN to identify whether the dressing was still in place, whether it was clean, dry and intact, whether additional dressing products had been applied and whether there were other complications at the insertion site (pain, tenderness, erythema, swelling, hardness, palpable cord, vein streak, or purulence). At PIV removal, the ReN collected the PIV dwell time, reason for device removal (device failure, routine replacement or completion of treatment), patient-reported pain or tenderness (11-point scale from 0 = no pain to 10 = maximum pain), redness, swelling and palpable cord (measured in centimetres from the insertion site), any adverse events relating to skin reactions associated with the dressing or securement products and the patient’s acceptability of the products. Feasibility outcomes (e.g. eligibility, recruitment, retention and attrition) were collected from enrolment screening logs and the de-identified electronic patient database.

### Statistical analysis

The data were exported to Stata 15 for analysis. An intention-to-treat analysis was used with the unit of analysis as one PIV per patient. Frequencies and proportions (%) were reported for categorical data. Means and standard deviations were reported for normally distributed continuous data. Median values and 25th and 75th percentiles were reported otherwise. Failure per group was expressed as rates per 1000 PIV days with 95% confidence intervals (CIs). The between-group incident rate ratio with 95% CI was calculated. A graph of the Kaplan–Meier survival function by group was generated and a log-rank test performed. Uni- and multivariable Cox regression modelling was used to assess the effect of a priori chosen patients and treatment characteristics (e.g. insertion site, dwell time and catheter gauge) based on variables suspected or known to be associated with the outcome in previous studies, as well as for group comparisons of failure, with hazard ratios (HRs) and 95% CIs calculated. To increase the sizes, categorical variables were re-coded where possible and reasonable to combine categories with similar effects. For example, all antibiotics were grouped into one variable. Based on the results of univariable analyses, covariates were deemed ineligible for multivariable analysis at *p* ≥ 0.20 or if the proportional hazards assumption test was significant. Correlations between covariates were checked. The final regression model was derived using manual backward/forward stepwise removal/addition of covariates at *p* < 0.05. The global proportional hazards assumption was tested, and the cumulative hazard of Cox–Snell residuals was plotted (not reported). Statistical significance was declared at *p* < 0.05. Missing values were not imputed. Data related to the feasibility outcomes were tabulated as percentages, reported descriptively and analysed against predetermined acceptability criteria: (i) more than 80% of patients screened will be eligible, (ii) more than 80% will agree to enrol, (iii) more than 90% will receive the allocated intervention and (iv) less than 5% of enrolled patients will be lost to follow-up.

## Results

Between October 2016 and July 2017, 300 patients were enrolled. At baseline, the groups had similar demographic and PIV characteristics (Table 1). Patients were typically males admitted from a surgical ward. The majority were overweight or obese. Most PIVs were inserted in the forearm and approximately half required multiple insertion attempts or were considered difficult insertions.

**Table 1:** Patient, insertion and device characteristics

	Control	Intervention	Total
Group size	150	150	300
Age (years)^a^	60.4 (17.1)	62.3 (18.6)	61.4 (17.9)
Males	88 (59%)	98 (65%)	186 (62%)
Weight appearance:			
Underweight	17 (11%)	19 (13%)	36 (12%)
Healthy weight	64 (43%)	60 (40%)	124 (41%)
Overweight	45 (30%)	52 (35%)	97 (32%)
Obese	24 (16%)	19 (13%)	43 (14%)
Inserted on dominant side	76 (51%)	72 (50%)	148 (51%)
Skin type (Fitzpatrick scale):			
Pale white	17 (11%)	20 (13%)	37 (12%)
White	116 (77%)	119 (79%)	235 (78%)
Light brown	15 (10%)	10 (7%)	25 (8%)
Moderate brown	1 (1%)	1 (1%)	2 (1%)
Deeply pigmented dark brown	1 (1%)	0 (0%)	1 (0%)
Skin integrity:			
Good	70 (47%)	58 (39%)	128 (43%)
Fair	66 (44%)	79 (53%)	145 (48%)
Poor	14 (9%)	13 (9%)	27 (9%)
Reason for admission:			
Medical	43 (29%)	43 (29%)	86 (29%)
Surgical emergent	27 (18%)	37 (25%)	64 (21%)
Surgical elective	81 (54%)	70 (47%)	151 (50%)
Infection at baseline	37 (25%)	43 (29%)	80 (27%)
Number of comorbidities:			
Zero	27 (18%)	15 (10%)	42 (14%)
One	17 (11%)	11 (7%)	28 (9%)
Two	10 (7%)	20 (13%)	30 (10%)
Three	16 (11%)	18 (12%)	34 (11%)
Four or more	80 (53%)	86 (57%)	166 (55%)
Wound at baseline	72 (48%)	80 (53%)	152 (51%)
Subsequent device per patient	142 (95%)	145 (97%)	287 (96%)
Device size:			
18 gauge	1 (1%)	1 (1%)	2 (1%)
20 gauge	86 (57%)	80 (53%)	166 (55%)
22 gauge	60 (40%)	64 (43%)	124 (41%)
24 gauge	3 (2%)	5 (3%)	8 (3%)
Placement:			
Cephalic vein	180 (60%)	85 (57%)	95 (63%)
Accessory cephalic vein	46 (15%)	25 (17%)	21 (14%)
Medial antebrachial vein	26 (9%)	16 (11%)	10 (7%)
Other vein	48 (16%)	24 (16%)	24 (16%)
Location:			
Posterior forearm	88 (59%)	87 (58%)	175 (58%)
Anterior forearm	25 (17%)	19 (13%)	44 (15%)
Wrist	28 (19%)	27 (18%)	55 (18%)
Hand	7 (5%)	13 (9%)	20 (7%)
Other	2 (1%)	4 (3%)	6 (2%)
Difficult insertion	56 (37%)	56 (37%)	112 (37%)
Multiple attempts at insertion	19 (13%)	25 (17%)	44 (15%)
Hairy skin:			
Yes, clipped	82 (55%)	79 (53%)	161 (54%)
Yes, unclipped	0 (0%)	2 (1%)	2 (1%)
No, unclipped	68 (45%)	69 (46%)	137 (46%)

Frequencies and column percentages shown, except where noted

^a^Mean and standard deviation shown

### Feasibility outcomes

**Fig. 2:**
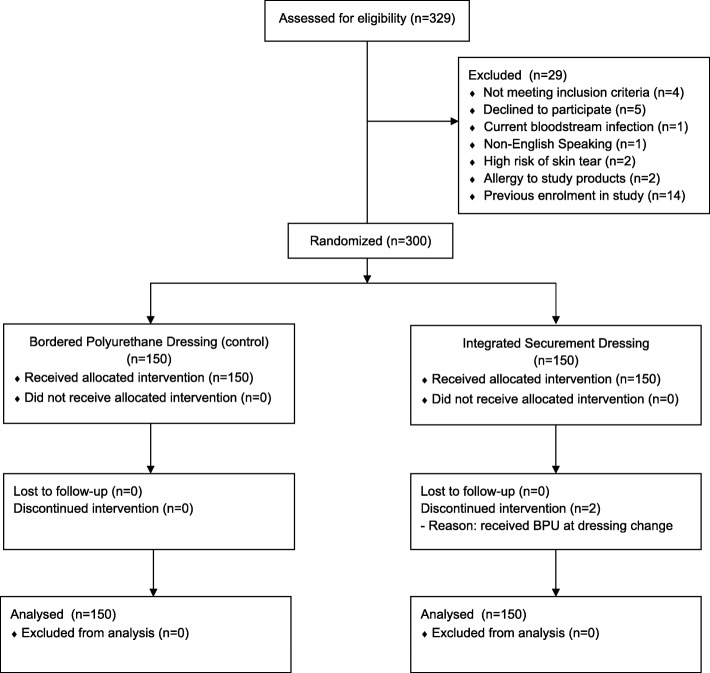
CONSORT flow chart. BPU bordered polyurethane dressing

Of the 329 participants screened, 91% were eligible to participate and therefore, the eligibility feasibility criterion of greater than 80% was met (Fig. 2). Furthermore, there was a high rate of willingness to participate, with only five patients (2%) refusing consent, typically the high treatment demands and complexity of care affected their readiness to engage in research. This met our consent feasibility criterion of greater than 80%. All participants received the allocated intervention at time of PIV insertion, resulting in 150 participants per study group. Subsequently two patients allocated to ISD (1%) were changed to standard care (BPU) on days 2 and 3, respectively, due to the ISD dressing lifting and the nurse replacing it with the incorrect product. Protocol adherence was, therefore, above the 90% required for feasibility. A single patient (0%) was lost to follow-up because they were transferred to another distant hospital facility; thus, the attrition feasibility criterion was met. Primary outcome data were available for the remaining patients. Patient satisfaction scores were provided by only 157 participants (52%). The reasons for this low response rate included: (i) discharge prior to the ReN being able to collect this score and (ii) the patient was unwilling or unable to provide a score at the time of device removal.

### PIV failure

**Fig. 3:**
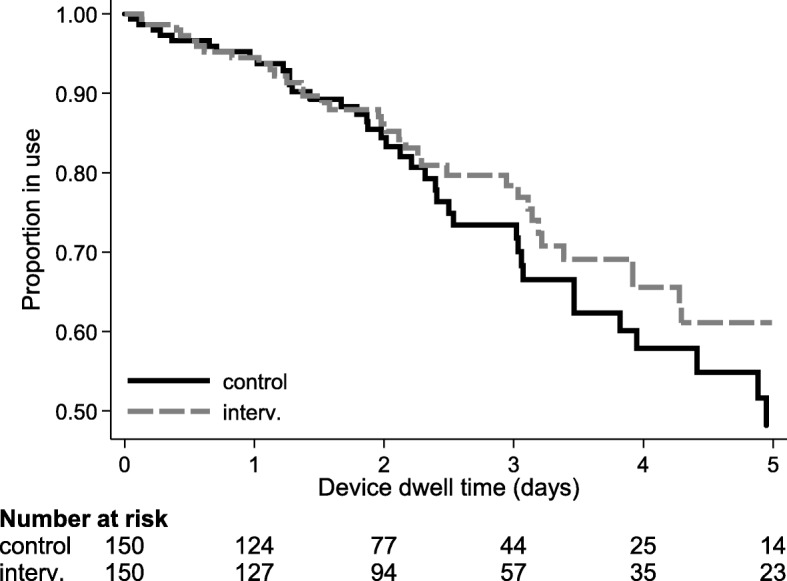
Kaplan–Meier curve of device failure

PIV failure was proportionally similar between groups, occurring in 43/150 (29%) BPU and 40/150 (27%) ISD patients (Table 2). There were 362 device-days studied in the BPU group and 430 device-days studied in the ISD group, with 119 and 93 failures per 1000 PIV days, respectively (incidence rate ratio 0.78, 95% CI 0.5–1.23, *p* = 0.137). PIV survival curves were not significantly different (*p* = 0.137) but displayed some separation by group from around 48 h of dwell (Fig. 3).

**Table 2:** Device and other outcomes, including time-to-event analysis (*n* = 300)

	Control	Intervention	*p* value
Primary outcome: device failure	43 (29)	40 (27)	0.699^a^
Dwell time (hours)^b^	57.9 (40.5)	68.8 (50.7)	
Dwell time (device-days)	362	430	
Incidence rate (95% CI, per 1000 device-days)	119 (88–160)	93 (68–127)	
Incidence rate ratio (95% CI)	Referent	0.78 (0.50–1.23)	0.137^c^
Why was the device removed?			
Treatment complete with complications	9 (6)	5 (3)	
Treatment incomplete with complications	37 (25)	39 (26)	
Treatment complete without complications	92 (61)	86 (57)	
Insertion of a CVAD	2 (1)	4 (3)	
Deceased	1 (1)	3 (2)	
Other	3 (2)	2 (1)	
Routine resite	5 (3)	9 (6)	
Admission to ICU	1 (1)	2 (1)	
Complication at removal:			
Occlusion, infiltration, extravasation, leakage	32 (21)	30 (20)	
Phlebitis, too painful to tolerate	9 (6)	8 (5)	
Partial dislodgement, accidental removal	6 (4)	6 (4)	
Other	4 (3)	5 (3)	
Serious adverse events:			
Death	2 (1)	4 (3)	
Positive blood culture	1 (1)	4 (3)	
ICU admission	1 (1)	2 (1)	
Phlebitis signs or symptoms^d^ (*n* = 163):			
Pain or tenderness ≥2 out of 10	3 (4)	5 (6)	
Erythema >0.5 cm	3 (4)	0 (0)	
Swelling >0.5 cm	4 (5)	5 (6)	
Hardness >2 cm	3 (4)	2 (2)	
Palpable cord	0 (0)	0 (0)	
Vein streak >1 cm	1 (0)	0 (0)	
Purulence	0 (0)	0 (0)	
Number of dressings used^e^	156 (1.04)	152 (1.01)	
Duration of first dressing (hours)	47.8 (27.1–73.3)	53.2 (28.8–94.0)	
Reason for dressing change:			
Dressing lifting	3	2	
Bleeding	2	0	
Unknown	1	0	
Protocol deviations: bordered polyurethane dressing	0	2	
Additional securement devices:^g^			
Elasticised tubular bandage	22%	28%	
Non-sterile tape	11%	15%	
Bandage	4%	0%	
Simple transparent dressing	2%	0%	
Fabric fixation tape	1%	0%	
Clean, dry and intact^g^	66%	83%	
Appearance of dressing:^g^			
Lifting slightly on the edges	12%	4%	
Dried blood at insertion site	3%	5%	
Lifting a great deal	4%	1%	
Blood leaking from the site	3%	1%	
Other	1%	0%	
Patient satisfaction (0 = worst, 10 = best; *n* = 157)^f^	9.0 (8.0–10.0)	9.0 (8.0–10.0)	

Frequencies and column percentages shown, unless otherwise noted

*CI* confidence interval, *CVAD* central venous access device, *ICU* intensive care unit

^a^Chi-squared test

^b^Mean and standard deviation

^c^Log-rank test

^d^Within 24 h of device removal

^e^Number and average number

^f^Median and 25th–75th percentiles

^g^Proportion of observations

### Secondary outcomes

There were no laboratory-confirmed local venous or primary bloodstream infections. Five patients (2%) had positive blood cultures. Of these, two (ISD group) were common commensals (likely skin contaminants), and three (one BPU and two ISD) were secondary bloodstream infections related to another source [19].

Occlusion and infiltration with or without leakage were the most common type of PIV failure, with a similar incidence in each group (32 BPU and 30 ISD). There was no difference in dislodgement incidence between the two groups (six BPU and six ISD) and a minimal difference in phlebitis (nine BPU and eight ISD). However, the average PIV dwell time was longer in the ISD group at 68.8 h than for the BPU group at 57.9 h, and daily site inspections observed a higher proportion of non-adhesion of a dressing in the BPU group (16%) compared with the ISD group (5%).

There were 12 skin reactions (six per group) to study products (4%). This included seven cases of mild itching (four ISD and three BPU), two cases of severe itching (BPU), one rash (BPU), one blister (ISD) and one skin tear (ISD). Eleven of the 12 skin reactions resolved quickly while the dressing was still in place, or shortly after device removal. The single skin tear was dressed with an appropriate adhesive and had almost resolved at the time of patient discharge from hospital 4 days later.

### Univariable and multivariable modelling for PIV failure

Significant risk factors for PIV failure (Table 3) in the univariable analysis were female gender, fair or poor skin integrity, 22/24 gauge PIV, insertion into the accessory cephalic or medial antebrachial vein and frequent PIV access (use). In addition, use of an elasticised tubular bandage or being obese compared with being underweight were significantly associated with fewer PIV failures. In the multivariable model, female gender, a wound at baseline, PIV hand insertion and more frequent PIV access were significantly (*p* < 0.05) associated with increased PIV failure. Admission with a surgical emergency and use of ISD (HR 0.51, 95% CI 0.29–0.89) were significantly associated with decreased PIV failure (*p* < 0.05).

**Table 3:** Cox multivariable regression

	Hazard ratio (95% CI)	
	Univariable	Multivariable
Intervention group (referent control)	0.72 (0.46–1.11)*	0.51 (0.29–0.89)**
Age (1-year increments)	1.01 (1.00–1.02)*	^
Female (referent male)	1.66 (1.08–2.57)**	1.83 (1.09–3.09)**
Weight category (referent underweight):		
Healthy	0.64 (0.34–1.21)*	§
Overweight	0.75 (0.39–1.44)	§
Obese	0.43 (0.19–0.99)**	§
Inserted on dominant side (referent no)	1.44 (0.92–2.25)*	§
Skin integrity: fair/poor (referent good)	1.64 (1.02–2.63)**	§
Surgical emergency admission (referent elective/medical)	0.56 (0.30–1.05)*	0.38 (0.17–0.86)**
Wound at baseline (referent no)	1.54 (0.98–2.42)*	2.17 (1.22–3.86)**
Device size 22/24 gauge (referent 18/20 gauge)	2.12 (1.37–3.29)**	§
Placement in accessory cephalic or medial antebrachial vein (referent other)	1.64 (1.04–2.59)**	§
Location: hand (referent forearm)	2.11 (0.96–4.62)*	2.79 (1.05–7.41)**
IV medication received:^a^		
Fluids	1.73 (0.92–3.26)*	§
Ceftriaxone	0.34 (0.06–1.81)*	§
Piperacillin/tazobactam	1.52 (0.86–2.70)*	§
Nothing	0.31 (0.08–1.16)*	^
Device in use^a^	3.55 (1.09–11.6)**	3.75 (1.09–12.8)**
Additional securements^a^:		
Elasticised tubular bandage	0.22 (0.08–0.64)**	§
Nil	2.15 (0.84–5.48)*	^

Covariates at univariate *p* ≥ 0.20 excluded; covariates breaching the proportional-hazards assumption at univariable analysis excluded

*CI* confidence interval, *IV* intravenous

*p < 0.20; **p < 0.05

^a^Variable set up as continuous variable between 0 (never observed) and 1 (observed at every check)

§Variable dropped at *p* ≥ 0.05 during multivariable model building; multivariable model: *n* = 227

^Excluded due to correlation with another covariate

## Discussion

Effective dressing and securement are likely to have an important role in reducing the incidence of PIV failure, which at present remains unacceptably high. The purpose of this pilot RCT was to compare the use of two dressing options for PIV to prevent all-cause failure and to explore feasibility measures for a larger definitive RCT. Feasibility outcomes were successfully met. The trial methods are appropriate for a multi-centre superiority two-arm RCT. As expected in this pilot trial, there was no statistically significant difference in the absolute proportion of all-cause PIV failure between BPU and ISD. However, the multivariable model demonstrated a halving of PIV failure associated with the ISD group compared with the BPU group. This confirms the need for further investigation in larger RCTs.

The complication-free dwell time observed in the ISD group compared with the BPU group was longer by 11 h. While this was not statistically significant, it can be regarded as clinically *important*, particularly since current guidelines support longer dwell PIVs if they remain symptom-free and are still needed [14, 21]. Current evidence indicates that hospitals with a clinically indicated replacement policy have an average PIV dwell time of 63–101 h [3, 22, 23], compared with those maintaining a routine replacement policy with an average PIV dwell of 58–70 h [3]. Moreover, many patients require multiple PIVs throughout their treatment, due to recurrent PIV failure [3]. Therefore, whilst clinically indicated replacement is a cost-saving measure [24], the high incidence of PIV failure prior to completion of therapy remains a barrier to longer functional dwell times. Further improvements, such as improved dressing and securement, are needed to improve patient outcomes. Compared with the PIV failure rates in the ISD and BPU groups of 27% and 29%, respectively, previous studies have reported PIV failure between 33% and 69% [4, 6, 7, 25]. It is unclear why the control and intervention groups each experienced lower than expected failure rates. However, a key contributing factor may have been the consistent use of an experienced vascular access nurse for all PIV insertions and initial dressing applications. While there is currently limited research exploring this influence, it has been suggested that PIVs inserted by vascular access nurses have lower failure rates compared with generalist inserters [26, 27]. This may have impacted not only on the appropriateness of the gauge size, location and insertion processes for study participants, but also the technique of dressing application.

Within our study’s multivariable modelling, female gender (compared with male gender) was significantly associated with PIV failure, a finding supported by other studies [28–32]. In particular, previous research has found females have a higher association with occlusion or infiltration and phlebitis [30, 31]*.* Consistent with previous studies, PIV insertion in the hand (compared with the forearm) was significantly associated with all-cause PIV failure, adding to the mounting evidence discouraging this insertion site [29, 30, 32]. Our study found that PIVs in use for a larger percentage of their dwell time (i.e. more injections and infusions) were statistically associated with a greater than threefold increase in PIV failure. This finding is supported by a large cohort study that found an increase in failure (HR 1.11–1.14) associated with a greater number of PIV accesses per day [31], possibly due to vein damage from frequent manipulation and delivery of fluids or medication. Our study also identified that the presence of a wound (at baseline) was associated with a greater than twofold increase of all-cause PIV failure. Barbut et al. [33] reported a significant association with skin lesions and the development of phlebitis. This suggests further investigation is needed to explore this finding.

Our multivariable analysis found that surgical emergency patients, compared with those admitted electively or as a medical admission, were associated with a lower risk of PIV failure. These surgical emergency patients (less likely to have been admitted for pre-existing conditions compared to their surgical elective and medical counterparts) likely had fewer previous PIVs that affected their vasculature [30, 34]. We also found that the ISD dressing significantly decreased PIV failure by half compared to standard care (BPU). This is an important clinical finding that may improve PIV care and patient outcomes. Future large trials are needed to confirm this finding. They should stratify by other factors that we have identified as significant (e.g. sex and admission category).

### Limitations

The main limitation was the inability to blind the study products from clinical and research staff due to their obvious nature and the need to provide PIV care. However, infection outcome assessments were blinded. This study provides valuable information, but as a pilot trial, it was not designed to provide definitive conclusions of the efficacy of the dressings. The use of experienced vascular access nurses to insert PIVs and apply dressings somewhat limits the generalisability of our results to clinical environments where generalist clinicians insert PIVs. However, the use of an experienced inserter in our study did ensure consistency of dressing applications, which was ideal for comparing the benefits and limitations for each group. Furthermore, apart from insertion, all other device use, practices and decision-making was as per the patient’s treating team and bedside nurses, which promotes the generalisability of our findings.

## Conclusion

The high rates of PIV failure reported in the literature indicate that our current dressings and securement products are inadequate. This study found the use of ISDs to dress and secure PIVs halved the risk of all-cause device failure in the multivariable analysis, compared to standard care BPU. This pilot trial has confirmed the need for a large multi-centre RCT to test these new innovative products.

## Abbreviations

BPU: Bordered polyurethane dressing
CI: Confidence interval
CVAD: Central venous access device
HR: Hazard ratio
ICU: Intensive care unit
ISD: Integrated securement device
IV: Intravenous
PIV: Peripheral intravenous catheter
RCT: Randomised controlled trial
ReN: Research nurse
SD: Standard deviation
